# Short-term recovery pattern of plasma fibrinogen after cardiac surgery: A prospective observational study

**DOI:** 10.1371/journal.pone.0201647

**Published:** 2018-08-03

**Authors:** Gabor Erdoes, Wulf Dietrich, Monika Pia Stucki, Tobias Michael Merz, Anne Angelillo-Scherrer, Michael Nagler, Thierry Carrel, Balthasar Eberle

**Affiliations:** 1 Department of Anesthesiology and Pain Medicine, Inselspital, Bern University Hospital, University of Bern, Bern, Switzerland; 2 Institute for Research in Cardiac Anesthesia, Munich, Germany; 3 Department of Intensive Care Medicine, Inselspital, Bern University Hospital, University of Bern, Bern, Switzerland; 4 Department of Hematology and Central Hematology Laboratory, Inselspital, Bern University Hospital, University of Bern, Bern, Switzerland; 5 Department of Cardiovascular Surgery, Inselspital, Bern University Hospital, University of Bern, Bern, Switzerland; University of Milano, ITALY

## Abstract

Low plasma fibrinogen level is common after cardiopulmonary bypass (CPB). Current substitution practice with fibrinogen concentrate generally follows a single measurement and cut-off values from the literature, whereas early postoperative endogenous fibrinogen kinetics is incompletely described and widely disregarded. The aim of this study was to determine the short-term recovery pattern of plasma fibrinogen after CPB weaning. Our hypothesis was that in the absence of surgical bleeding, CPB-induced hypofibrinogenemia would resolve spontaneously and predictably within a few hours. In a prospective, observational study of 26 patients undergoing conventional CPB (cCPB) or minimally invasive extracorporeal circulation (MiECC), Clauss fibrinogen level (C-FIB) was determined at 10 closely spaced time points after protamine administration. Primary endpoint was the time to recovery of post-CPB fibrinogen levels to ≥1.5 g/L. C-FIB reached its nadir after protamine administration corresponding to 62 ± 5% (mean ± SD) of the baseline level after cCPB and 68 ± 7% after MiECC (p = 0.027 vs. cCPB). C-FIB recovered spontaneously at a nearly constant rate of approximately 0.08 g/L per hour. In all patients, C-FIB was ≥1.5 g/L at 4 hours and ≥2.0 g/L at 13 hours after CPB weaning. Following cardiac surgery with CPB and in the absence of surgical bleeding, spontaneous recovery of normal endogenous fibrinogen levels can be expected at a rate of 0.08 g/L per hour. Administration of fibrinogen concentrate triggered solely by a single-point measurement of low plasma fibrinogen some time after CPB is not justified.

## Introduction

Fibrinogen reaches critically low levels during acute bleeding. [1] A close association has been reported between hypofibrinogenemia and severe post-cardiopulmonary bypass (CPB) bleeding. [2–6] Consequently, monitoring fibrinogen levels and function and algorithm-based substitution with fibrinogen concentrate or fresh frozen plasma (FFP) is recommended. [2,3,7–11] However, variation in the recommended tests, their exact timing, and published thresholds for substitution with exogenous fibrinogen sources render fibrinogen substitution practices quite inconsistent at present. Some authors have even challenged the indication for fibrinogen substitution. [12]

Fibrinogen is also an acute phase protein with plasma levels that rapidly increase in response to tissue trauma or inflammation. [13] As early as one day after CPB surgery, normal or even supranormal fibrinogen levels are regularly observed and induce a prothrombotic situation. [14–16] Sparse data are available describing the spontaneous development of endogenous plasma fibrinogen during the first few hours after CPB, whereas the early pharmacokinetics and effects of exogenous fibrinogen concentrate are much better documented. [17]

Reduction of fibrinogen levels after CPB is a physiologically expectable phenomenon. However, recommendations for exogenous fibrinogen substitution are often based on an early plasma fibrinogen measurement at only one point after CPB weaning. [11] Thus, knowledge of spontaneous plasma fibrinogen recovery during the initial hours after CPB appears to be a basic requirement for rational post-CPB coagulation therapy and specifically for factor substitution. Particularly in patients with mild post-CPB hypofibrinogenemia, this knowledge may justify watchful waiting and prevent costly overtreatment with exogenous fibrinogen sources.

The primary aim of this study was to closely monitor the time course of endogenous plasma fibrinogen during the first 24 hours after cardiac surgery with CPB. Second, we quantified the influence of CPB-induced hemodilution on endogenous fibrinogen decline and recovery by comparing conventional CPB (cCPB) and CPB with a reduced priming volume (minimally invasive extracorporeal circulation, MiECC) in a prospectively planned sub-analysis. Our hypothesis was that hypofibrinogenemia associated with hemodilution rather than bleeding would resolve spontaneously within a few hours after weaning from CPB.

## Methods

This prospective, observational study investigated patients who underwent cardiac surgery with CPB between November 12, 2015, and July 29, 2016, at a tertiary referral hospital. Ethical approval (No. 193/15) was provided by the Cantonal Ethical Committee on September 22, 2015. The study protocol was registered with the US National Institutes of Health (NCT02605330, www.ClinicalTrials.gov). All study measures were performed according to the Declaration of Helsinki. The manuscript adheres to the statement of the EQUATOR Network (www.equator-network.org).

Elective adult patients scheduled for cardiac surgery with CPB for either isolated coronary artery bypass grafting (CABG), isolated aortic valve replacement (AVR), or ascending aorta replacement (AAR) with hypothermic circulatory arrest (HCA) were studied. The inclusion criteria were patients above 18 years of age who signed an informed consent. The exclusion criteria were emergency or redo surgery, anticoagulant medication other than acetylsalicylic acid within 14 days preceding surgery, a preoperative plasma fibrinogen concentration below the lower limit of the institutional laboratory reference of 1.75 g/L, known coagulation disorders, a hemoglobin level less than 100 g/L, renal insufficiency with an estimated glomerular filtration rate <60 mL/min/1.73 m^2^, affiliation with Jehovah’s Witnesses, participation in another trial, or intraoperative supplementation with any plasma product.

All patients received general anesthesia in accordance with institutional standards. Perioperative monitoring included American Society of Anesthesiologists standard monitoring, invasive arterial and central venous pressure, transesophageal echocardiography, and processed EEG. Cardiac surgery was performed through median sternotomy. CABG was performed exclusively using MiECC (Maquet AG, Rastatt, Germany). For the AVR and AAR procedures, a cCPB circuit (Maquet AG) was employed. The main differences between MiECC and cCPB were as follows: 1) the total amount and composition of the priming volume (MiECC: 600 mL of lactated Ringer’s solution and 5000 IU of unfractionated heparin; cCPB: 1000 mL of lactated Ringer’s solution, 500 mL of hydroxyethyl starch (HES, 130/0.4), and 10000 IU of heparin); 2) the type of driving pump (MiECC: centrifugal pump and cCPB: roller pump); and 3) the processing method used for shed blood and residual pump blood. In MiECC, shed blood was recovered and filtered into a reservoir using an automated suction device (Cardiosmart AG, Muri, Switzerland) designed to minimize air entrainment and then returned to the venous line. In cCPB, shed blood was filtered and returned to the venous reservoir via a roller pump. Neither the MiECC nor the cCPB tubing was heparin-coated. The extracorporeal flow rate was set to 2.0 L/min/m^2^ of body surface area (MiECC) or to 2.4 L/min/m^2^ (cCPB). The nasopharyngeal temperature was maintained between 32°C and 36°C in CABG and AVR. In AAR, hypothermia was induced to a bladder temperature of 28°C. Cardioplegic arrest was achieved using a single dose of 100 mL of crystalloid cardioplegia (Cardioplexol^®^, Bichsel AG, Interlaken, Switzerland), and maintained by high potassium cold blood cardioplegia (Buckberg Solution, Bichsel AG, Switzerland). The anticoagulation regimen consisted of an IV bolus of heparin (MiECC: 400 IU/kg and cCPB: 500 IU/kg) prior to aortic cannulation, with the aim of an activated clotting time >480 sec (kaolin-activated HR-ACT, ACT II Plus, Medtronic Inc., Minneapolis, MN, USA). All patients received a bolus of tranexamic acid (30 mg/kg) followed by continuous infusion of 15 mg/kg/hour until sternal closure. No topical hemostasis techniques (e.g., agents containing fibrinogen or thrombin) were used. After weaning from CPB, heparin was neutralized with protamine chloride in a 1:1 ratio to the initial heparin dose. In MiECC, residual pump blood was completely reinfused into the patient prior to heparin neutralization. In cCPB, residual blood was processed (Medtronic Autolog, Medtronic Inc., Minneapolis, MN, USA) and re-transfused as an autologous red cell concentrate.

According to Ranucci, an increased *a priori* risk of bleeding was assumed if the duration of CPB was expected to exceed 90 minutes, patient age was >65 years, or the serum creatinine level was >1.36 mg/dL. [18] Perioperative bleeding was defined according to Dyke [19] and rated as severe in case of delayed sternal closure, re-exploration, pericardial tamponade, postoperative chest tube drainage of more than 1000 mL/12 hours and if transfusion of more than 5–10 units of packed red blood cells (RBC) or FFP was necessary within the first postoperative day.

### Data collection

Data were stored on a departmental research platform (Labkey, Seattle, WA, USA). Fibrinogen was measured using the Clauss method (Clauss fibrinogen, C-FIB) and using fibrin-based thrombo-elastometry (FIBTEM-MCF assay, ROTEM^®^, TEM International GmbH, Munich, Germany). Whole blood was serially sampled in citrated plastic containers (Monovette, Sarstedt AG, Nümbrecht, Germany) at defined time points as follows: baseline level (pre-CPB value) before anesthesia induction; *AodX*, five minutes after aortic declamping; *Protamine*, five minutes after protamine administration; and *T1*, *T2*, *T3*, *T4*, *T6*, *T8*, *T12*, *T18*, and *T24* at 1, 2, 3, 4, 6, 8, 12, 18, and 24 hours after protamine administration, respectively. All samples were immediately sent to the laboratory, pre-heated to 37°C, and analyzed within 15 minutes. C-FIB was determined using the Multifibren U reagent (Dade Behring, Liederbach, Germany) on a BCS-XP coagulometer (Siemens Healthcare, Marburg, Germany). For FIBTEM, tissue factor was used as the activator, and cytochalasin D was added for platelet inhibition. To minimize user-dependent variability, all coagulation and viscoelastic testing was performed by the hemostasis laboratory.

### Study endpoints

The primary endpoint was the post-CPB recovery time of the Clauss fibrinogen concentration (i.e., from 5 minutes after protamine administration to the time point when the Clauss fibrinogen levels surpassed a threshold of ≥1.5 g/L). The secondary endpoints included the association of hypofibrinogenemia with hemodilution and hypothermia, the post-CPB fibrinogen nadir, the time to recovery to the pre-CPB value, and recovery patterns of FIBTEM-MCF.

### Statistical analysis

Demographic and procedure-related variables were analyzed with contingency table analysis or nonparametric ANOVA as appropriate (Sigma Plot for Windows, version 12.2; Systat Software Inc., Germany). A proportional decrease in C-FIB and hemoglobin after CPB was quantified as the ratio of the respective concentration measured at *Protamine* to the ratio measured at baseline. Due to the non-normal distributions of the ratios and the log differences, this analysis was based on a Wilcoxon signed rank test, and comparisons were made using the Mann-Whitney test. Statistical analysis of the post-CPB course of C-FIB was performed in R, version 3.3.0. [20] C-FIB data pertaining to surgery type were fitted for separate linear mixed-effects models, with time as a fixed-effect covariate and a random effect for each patient. The random effects for a particular patient were the deviations in the intercept and slope of that patient's C-FIB time trend. The two random effects were considered non-correlated, which was confirmed by model comparison using the likelihood ratio test. The linear regression model was fitted to un-transformed and log-transformed data, reporting Wald-type confidence interval. Additionally, the influence of the baseline C-FIB level, the estimated preoperative weight-based plasma volume and the fluid administered during CPB on C-FIB at *Protamine* were assessed using linear regression models. Since the primary endpoint of this study was purely descriptive, a sample size calculation was not performed. P values <0.05 were considered significant.

Data are presented as numbers (percentages), means ± standard deviations, medians (25^th^; 75^th^ percentiles) or minimum and maximum (min/max) values.

## Results

**Fig 1:**
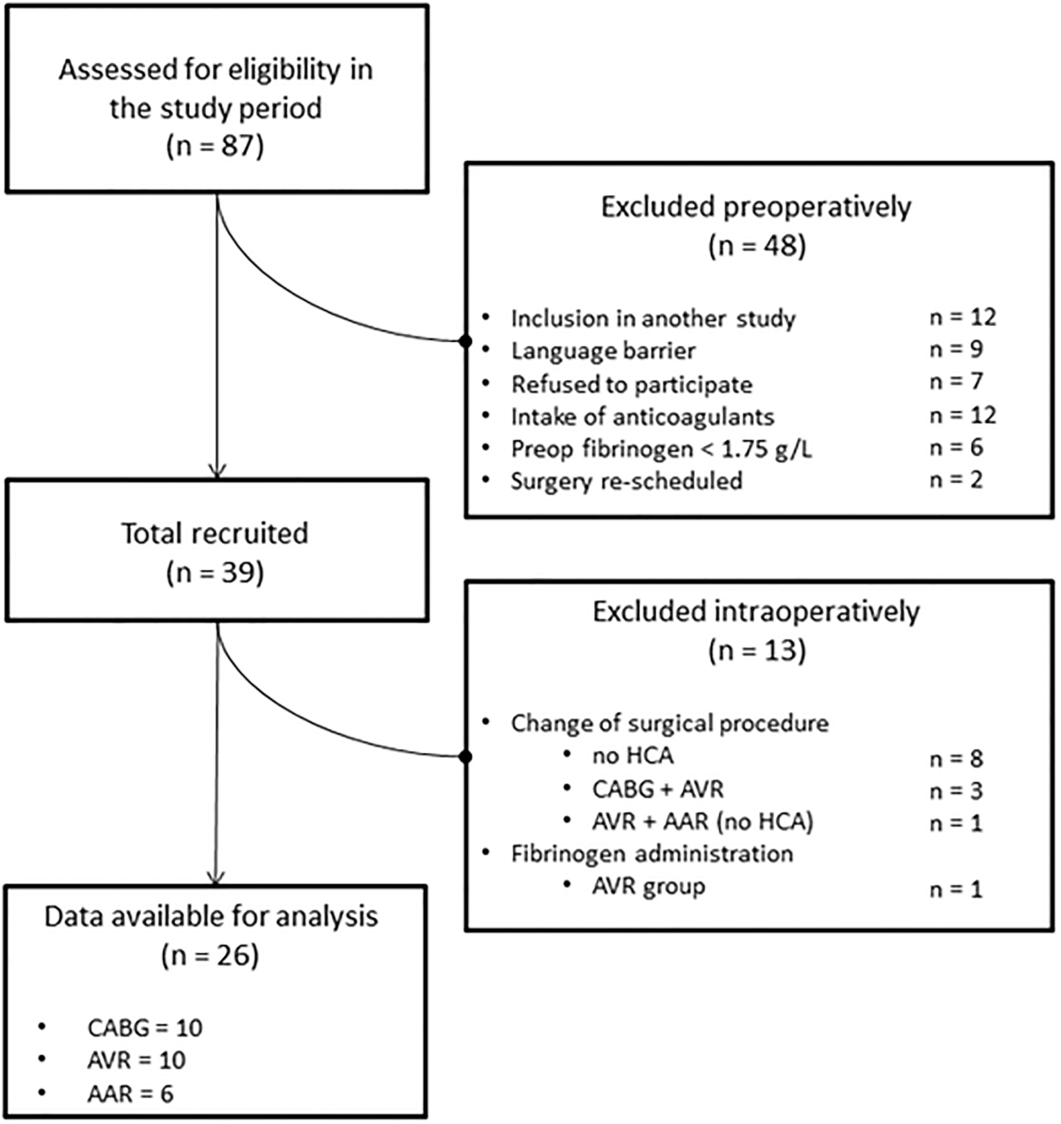
Patient flow diagram. CABG = coronary artery bypass grafting; AVR = aortic valve replacement; AAR = ascending aorta replacement; HCA = hypothermic circulatory arrest. n indicates number of patients.

Overall, 87 patients were assessed for eligibility. Forty-eight patients were excluded preoperatively and 13 intraoperatively. Twenty-six patients fulfilled the inclusion criteria and were analyzed (Fig 1).

Demographic and preoperative data are listed in Table 1. The procedural data and basic outcomes are summarized in subsequent sections of this table. Due to the use of MiECC for perfusion, CABG patients were exposed to the smallest CPB priming volume, the lowest amount of Ringer’s infusion, and no HES during CPB. Overall, 10 of the 26 patients (38%) fulfilled the preoperative criteria for an increased bleeding risk according to the definition of Ranucci [18]

**Table 1:** Patient demographics, procedural data and outcomes.

Procedural data					
CPB duration (min)	80 (62;115)	69 (57;92)	73 (61;101)	130 (111;141)	0.008
Cross clamp time (min)	52 (38;75)	42 (34;60)	49 (37;67)	82 (62;105)	0.04
HCA duration (min)	---	---	---	12 (10;15)	---
Lowest temperature (°C)	33 (31;34)	33 (33;34)^†^	34 (33;34) ^††^	27 (24;28) ^††^	<0.001
CPB prime (ml)	---	600	1500	1500	<0.001
Tranexamic acid (g)	2.2 (1.9;2.4)	2.2 (1.7;2.8)	1.9 (1.7;2.2)	2.2 (2.0;4.3)	0.11
Heparin dose (kIU)	39 (35;45)	35 (30;48)	40 (34;45)	42 (39;48)	0.06
On CPB, infused volume					
RBC (U)	0/2	0/0	0/0	0/2	0.03
Lactated Ringer’s (ml)	1957 (1513;2412)	1402 (1215;1950)	2019 (1696;2497)	2423 (2051;2846)	0.004
HES 130/0.4 (ml)	0/500	0/0	0/500	0/500	<0.001
Pre-, post-CPB volume					
RBC (U)	0/0	0/0	0/0	0/0	1.00
FFP (U)	0/0	0/0	0/0	0/0	1.00
Platelets (U)	0/0	0/0	0/0	0/0	1.00
ARBC (ml)	216 (0;347)	0 (0;0)	310 (219;430)	348 (252;538)	<0.001
Lactated Ringer’s (ml)	2074 (1275;3047)	2700 (1821;3071)	2075 (1795;2746)	1688 (1123;2320)	0.25
HES 130/0.4 (ml)	0/0	0/0	0/0	0/0	1.00
ICU, infused volume					
RBC (U)	0/4	0/2	0/2	0/0	0.48
FFP (U)	0/0	0/0	0/0	0/0	1.00
Platelets (U)	0/0	0/0	0/0	0/0	1.00
Lactated Ringer’s (ml)	3489 (2145;5978)	3497 (2402;5979)	4262 (2047;6684)	3173 (1660;4006)	0.47
HES 130/0.4 (ml)	0/0	0/0	0/0	0/0	1.00
Drainage, 6 h (ml)	180 (150;275)	262 (150;312)	150 (120;362)	165 (142;187)	0.31
Drainage, 12 h (ml)	300 (230;470)	412 (295;512)	250 (210;575)	260 (200;330)	0.14
Drainage, 24 h (ml)	525 (397;735)	712 (537;762)	510 (387;812)	355 (250;465)	0.02
ICU LOS (days)	0.9 (0.9;1.0)	0.9 (0.8;1.0)	1.0 (0.9;1.1)	0.9 (0.9;0.9)	0.06
In-house mortality, n (%)	0 (0)	0 (0)	0 (0)	0 (0)	1.00
	**All Patients** **(n = 26)**	**CABG** **(n = 10)**	**AVR** **(n = 10)**	**AAR** **(n = 06)**	**P** Value*
Demographics					
Male sex (n, %)	20 (77)	8 (80)	8 (80)	4 (66)	0.80
Age, y	67 ± 11	67 ± 11	70 ± 12	61 ± 6	0.12
BMI (kg/m^2^)	27 (24;29)	28 (24;31)	27 (24;29)	28 (25;31)	0.68
CAS > 70%	2 (8)	1 (10)	1 (10)	0 (0)	0.81
COPD (n, %)	5 (19)	3 (30)	0 (0)	2 (33)	0.15
Diabetes (n, %)	2 (8)	2 (20)	0 (0)	0 (0)	0.23
Preoperative laboratory					
Hemoglobin (g/L)	137 (129;147)	135 (129;147)	142 (131;149)	131 (115;140)	0.39
Platelets (G/L)	190 (175;232)	187 (182;225)	174 (148;225)	224 (195;265)	0.14
Fibrinogen (g/L)	2.8 (2.5;3.1)	3.0 (2.5;3.6)	2.9 (3.1;2.1)	2.7 (2.4;2.9)	0.47
FIBTEM-MCF (mm)	19 (16;21)	20 (17;21)	18 (15;22)	19 (17;20)	0.78

CABG = coronary artery bypass grafting; AVR = aortic valve replacement; AAR = ascending aorta replacement; BMI = body mass index; CAS = carotid artery stenosis; COPD = chronic obstructive pulmonary disease; FIBTEM-MCF fibrin-based thromboelastometry; CPB = cardiopulmonary bypass; HCA = hypothermic cardiocirculatory arrest; RBC = red blood cells; FFP = fresh frozen plasma; ARBC = autologous red blood cells; HES = hydroxyethyl starch; ICU = intensive care unit; ICU LOS = intensive care unit length of stay; KIU = kilo international units; U = unit (corresponds to 275 mL);

^†^nasopharyngeal temperature;

^††^core temperature. Data are numbers (percent), mean ± standard deviation, median (25^th^; 75^th^) or minimum and maximum (min/max) values.

*indicates comparison of CABG/AVR/AAR with contingency table analysis or Kruskal-Wallis one-way ANOVA on Ranks where appropriate.

The transfusion incidence with allogeneic red blood cell concentrates (RBC) was 3/26. One patient received RBC during CPB, and another two patients were transfused during their intensive care unit (ICU) stay. None of the patients received FFP, platelets, fibrinogen, or any other factor concentrate (exclusion criteria). During their ICU stay, the cumulative mediastinal drainage loss after 6 and 12 hours amounted to medians of 180 and 300 mL, respectively. Thus all patients, including those assumed pre-operatively to be at an increased risk of bleeding, [18] were in postoperative bleeding categories 0 (insignificant) or 1 (mild). [19] After only 24 hours, the CABG/MiECC patients had accumulated a slightly higher drainage loss than the other groups (p = 0.02 vs. cCPB). This was because one patient in the CABG group experienced transient hemodynamic instability and was treated with transfusion of 2 units of RBC postoperatively. No differences between groups were found regarding transfused blood products, incidence of new infections, thrombotic events, or in-house mortality.

### Primary endpoint

**Fig 2:**
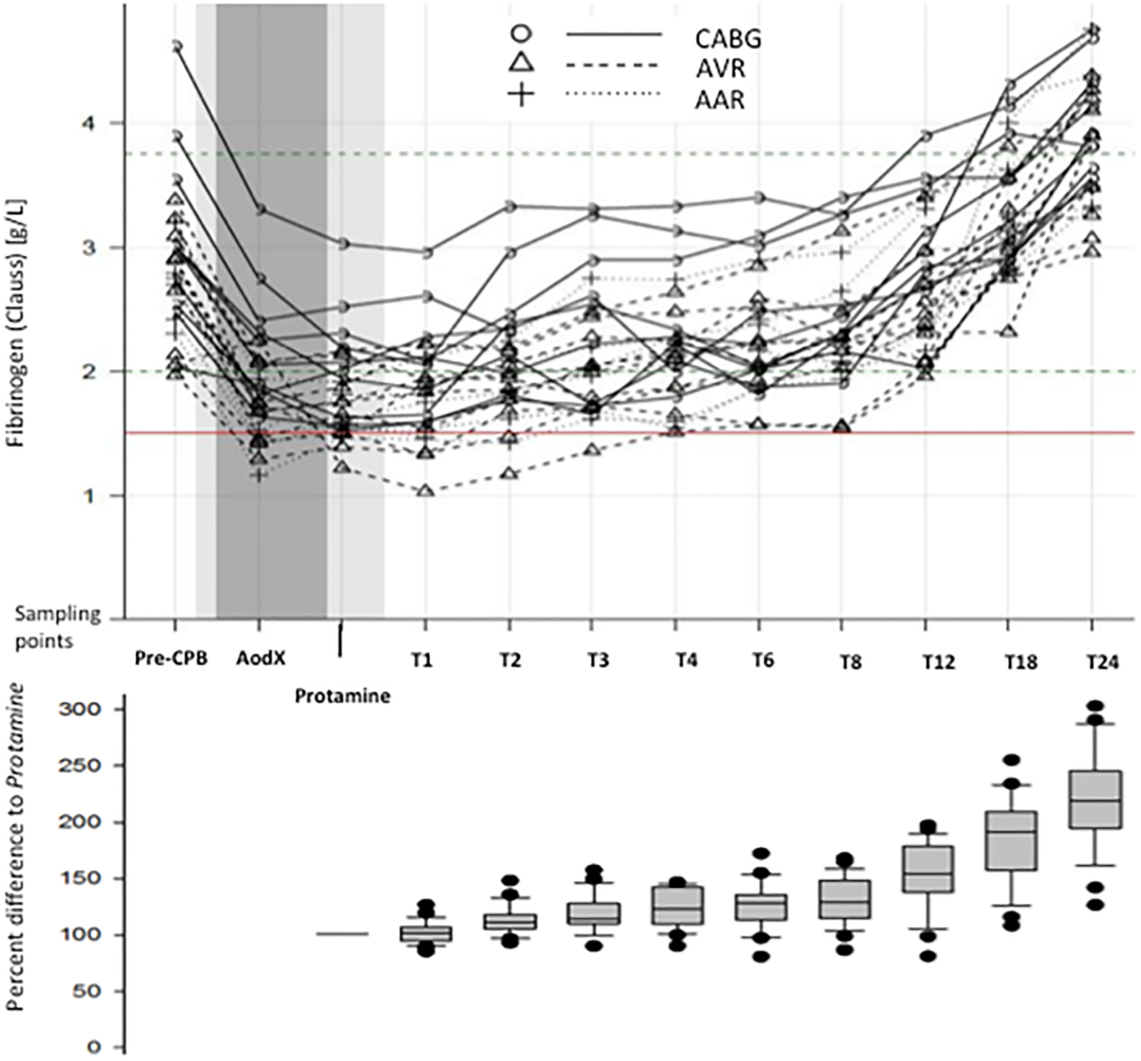
Fibrinogen concentration at the sampling points and changes in relation to time point *Protamine*. Clauss fibrinogen concentration in all patients during the observation period (upper graph), and relative post-CPB change as percent difference from levels at time point *Protamine* (lower graph). In the upper graph the light grey shaded area indicates the period of surgery. Time on CPB is shaded dark grey. The red horizontal line indicates Clauss fibrinogen at 1.5 g/L. Note that true time intervals increase between sequence points *T1* and *T24*.

The perioperative nadir of the C-FIB was observed five minutes after protamine administration (*Protamine)*, at which time the C-FIB had decreased to 64.3 ± 7% of baseline (Fig 2). Fibrinogen was significantly better preserved with MiECC (at *Protamine*: MiECC, 67.8 ± 7% vs. cCPB, 62.1 ± 5%; p = 0.027).

**Fig 3:**
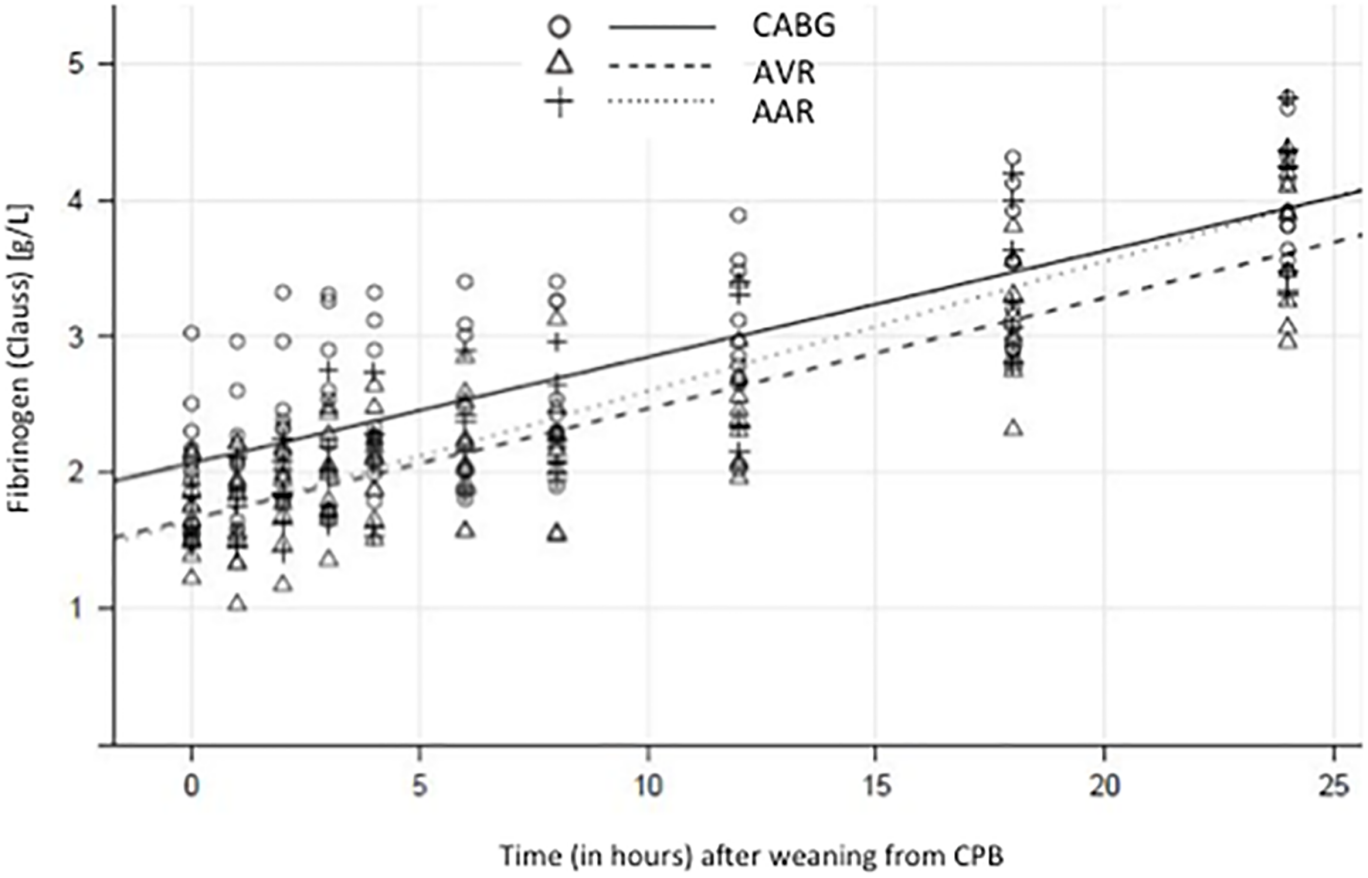
Clauss fibrinogen increase after weaning from CPB, according to surgery type. Clauss fibrinogen measurements over time, starting at the time point *Protomine*. The comprehensive plots show all patients, with the three types of surgery represented by different symbols. Predictions based on the fixed effects of the three respective linear mixed-effects models are superimposed.

C-FIB significantly increased throughout the subsequent 24 hours (Fig 3). Patients undergoing surgery on MiECC had significantly higher endogenous plasma fibrinogen levels during the first four hours after heparin reversal than patients perfused with cCPB (p = 0.022). The post-CPB recovery of the C-FIB over time was fitted with linear regression models. The mean Clauss fibrinogen (all patients) increased after *Protamine* at a nearly constant rate of 0.083 g/L per hour (95% CI, 0.076 to 0.091; R^2^ = 0.988, p <0.0001), corresponding to an hourly recovery of 5% of the fibrinogen level present at *Protamine*. Surgery-specific rate estimates of the Clauss fibrinogen increase were 0.078 g/L per hour for CABG (0.061 to 0.095), 0.081 g/L per hour for AVR (0.070 to 0.094), and 0.095 g/L per hour for AAR (0.076 to 0.115); no between-group differences were observed (p = 0.371).

The post-CPB recovery times of the C-FIB levels (i.e., from *Protamine* to the time point at which the levels surpassed the thresholds of ≥1.5 g/L or ≥2.0 g/L) were determined from the individual datasets. Post-CPB, 5/26 patients (19%) had fibrinogen levels <1.5 g/L, and 18/26 (69%) patients had levels <2.0 g/L. All patients had levels above 1.5 g/L within 4 hours and above 2.0 g/L within 13 hours after heparin reversal.

### Secondary endpoints

Between the baseline measurement at induction of anesthesia and the *Protamine* time point, the C-FIB course differed depending on the type of surgery and the type of CPB. The smallest decrease to a median of 2.15 g/L (1.86;2.39) was observed for CABG at the time of heparin reversal, whereas the C-FIB remained at 1.5 g/L or higher in all these patients. The AVR and AAR showed lower nadirs of the median 1.71 g/L (1.51;1.98) and 1.46 g/L (1.40;1.51), respectively (AVR vs. AAR: n.s.). Thus, at the end of CPB, the fibrinogen levels were significantly less depressed for MiECC than for cCPB (p = 0.001). Recovery to the C-FIB baseline occurred within a median time of 11.2 hours (9.2;16.5) from heparin reversal, with no significant differences between the types of surgery (p = 0.860) or CPB (p = 0.854). CPB-related hemodilution from an asanguineous prime and fluid administered during CPB reduced the hemoglobin concentration (Hb) in all patients to 76 ± 6% of the baseline at *Protamine*, with no difference between MiECC and cCPB (p = 0.542). During the same period, the C-FIB de-creased to 64 ± 7% of the baseline (p<0.001 vs. Hb).

The C-FIB nadir at *Protamine* was mainly determined by the fibrinogen levels prior to CPB and to a lesser degree by the preoperative weight-based patient plasma volume. An asanguineous fluid load during CPB had a weak negative effect (Table 2).

**Table 2:** Patient- and CPB-related factors influencing clauss fibrinogen levels and FIBTEM-MCF at *Protamine*.

		R	95% CI	p-value
Clauss fibrinogen	Fibrinogen at <Pre-CPB>	0.76	0.56 to 0.96	< 0.0001
	Estimated plasma volume	0.28	0.01 to 0.56	0.045
	Fluid administered on CPB	-0.09	-0.18 to 0.00	0.04
FIBTEM-MCF	FIBTEM-MCF at <Pre-CPB>	0.91	0.64 to 1.18	< 0.0001
	Estimated plasma volume	0.21	-0.23 to 0.65	0.33
	Fluid administered on CPB	-0.15	-0.27 to -0.03	0.015

Linear regression models, log-transformed data. Estimated coefficients (R), 95% Wald-type confidence intervals (CI), and p-values from a *t* distribution.

FIBTEM-MCF patterns prior to, during and after CPB were generally correlated with those of Clauss fibrinogen. Post-CPB, 4/26 patients (15%) had a FIBTEM-MCF <10 mm. At heparin reversal, the median FIBTEM-MCF was 14 mm (9;21). Overall, FIBTEM-MCF had a larger variance but was significantly correlated with Clauss fibrinogen (r = 0.810; p<0.001). Among the three types of surgery, FIBTEM-MCF in CABG remained significantly higher in the post-CPB period than in AVR (difference of means, 2.9 mm) and AAR (difference of means, 2.0 mm) (each p<0.001 vs. CABG; AVR vs. AAR, difference of means, 0.9 mm, p = 0.115). Consequently, MiECC was consistently associated with a higher FIBTEM-MCF than cCPB (difference of means, 2.6 mm, p< 0.001). FIBTEM-MCF at heparin reversal was significantly correlated with baseline FIBTEM-MCF and was negatively associated with asanguineous fluid load during CPB (Table 2).

The sample size did not have sufficient power to discriminate the effects of CPB duration or the lowest temperature on post-CPB C-FIB and FIBTEM-MCF. Clauss fibrinogen and FIBTEM-MCF after *Protamine* were not correlated with postoperative chest drain output.

## Discussion

The main finding of this prospective study in adults undergoing elective on-pump cardiac surgery for either isolated coronary artery bypass grafting, isolated aortic valve replacement, or ascending aorta replacement with hypothermic circulatory arrest (HCA) is that endogenous plasma fibrinogen increases from its nadir at the time of heparin reversal at a fast and nearly constant rate of approximately 0.08 g/L per hour during the subsequent 24 hours. All patients had fibrinogen levels at or above 1.5 g/L within 4 hours and at or above 2.0 g/L within 13 hours of heparin reversal. Thus, the results confirm our initial hypothesis that hypofibrinogenemia resolves spontaneously within a few hours after CPB and heparin reversal.

Previous studies in cardiovascular surgery have reported that fibrinogen levels initially fall to nearly 50% after CPB [4,14,21–24] and recover to baseline or above after the first postoperative day, independent of whether an exogenous fibrinogen concentrate has been administered. [16,17,25] The typical decrease in plasma fibrinogen has been mainly attributed to hemodilution [22,26] as a consequence of using asanguineous fluid to prime the CPB circuit and for volume resuscitation. [27] Our results confirm the role of CPB-induced hemodilution as a major contributor to immediate post-CPB hypofibrinogenemia. Consequently, during the initial four hours after heparin reversal, we observed better preservation of plasma fibrinogen with MiECC than cCPB due to less hemodilution from the prime.

Fibrinogen substitution is recommended for bleeding patients. [11] However, predicting individual patients’ risks of significant or even severe non-surgical bleeding for the first few hours following CPB has proven difficult both pre-operatively and at CPB weaning. [16,18] This difficulty occurs because clinical bleeding assessments remain at least partially subjective and arbitrary and do not produce unequivocal results even with substantial efforts towards standardization. [16,25] Additionally, the universal definition of perioperative bleeding relies on longitudinal assessment of cumulative chest drainage, which is necessarily retrospective, and on treatment interventions, which are not guided by universal protocols. [19] These difficulties may explain why early laboratory-guided administration of fibrinogen concentrate in randomized controlled studies of cardiovascular patients at presumed high risk of bleeding has not been proven effective in terms of a clinically relevant reduction of blood loss, transfusion, or adverse outcomes to date. [16,25] Our data demonstrate that, if fibrinogen substitution is either laboratory-guided or point-of-care (POC)-guided, the time point of measurement is, due to the fast recovery rate, essential. There is a marked difference in the results depending on whether measurements are performed just after CPB or at ICU admission. The exact time point of measurement is not delineated in all studies.

Numerous studies have been performed in vitro, in animals, and in humans with the aim of preventing or treating coagulopathies associated with the dilution or loss of endogenous fibrinogen by exogenous supplementation of fibrinogen concentrate. These studies have generally succeeded in increasing the plasma fibrinogen levels for a short time but have not consistently reduced postoperative blood loss or allogeneic transfusion. [10,16,28–35] In a randomized trial that targeted a plasma fibrinogen level of 2.5 g/L in high-risk cardiac surgery patients, administration of fibrinogen concentrate in bleeding patients did not result in a significant reduction in intraoperative blood loss compared to a placebo.[25] Similarly to our findings, the nadir of plasma fibrinogen occurred at the end of CPB, with a spontaneous recovery towards ICU admission and at 24 hours thereafter even in the group without exogenous fibrinogen substitution. The mean plasma fibrinogen was 1.7 g/L at the end of CPB and 3.1 g/L 24 hours after placebo infusion, without an increased bleeding tendency compared to that of the group receiving fibrinogen concentrate. [25] Similarly, Rahe-Meyer et al. described a fibrinogen concentration of 1.9 g/L at skin closure in their saline placebo group, and recovery to 3.2 g/L within 24 hours. No increased bleeding was seen in patients who did not receive the fibrinogen concentrate compared to those in the control group. [16] Notably, nearly 75% of patients in both of these RCTs would have been classified *post hoc* as having only insignificant or mild bleeding according to the universal definition [19] based on the 12-hour chest drainage loss data (IQR), regardless of the treatment allocation. Similarly, our study sample also included patients with a predicted high bleeding risk (38%), but 25/26 did not even bleed moderately (class 0 or 1 only). [19] No FFP or platelet concentrate was required in our series, and only two patients (8%) received two units of allogeneic RBC each during their ICU stay. The C-FIB nadir level after protamine administration was not predictive of the subsequent chest drain output, with a median of 300 mL in the first 12 postoperative hours. This finding supports the notion that hemodilution-induced nadirs of Clauss fibrinogen similar to those observed in our study do not justify fibrinogen substitution *per se*.

Ample evidence shows a statistical association between low plasma fibrinogen and increased bleeding after cardiac surgery. [2–6] However, this association is neither always causal nor always unidirectional. Additionally, fibrinogen concentrate substitution did not ameliorate bleeding tendency. Surgical or thrombopenic bleeding may cause or aggravate hypofibrinogenemia and vice versa, and mutual causation may vary during a difficult postoperative course. Therefore, recent guidelines for severe post-CPB bleeding recommend maintenance of the C-FIB in a range from 1.5 to 2.0 g/L.,[11]

In cases of severe hemodilution, an even higher level of ≥2.0 g/L is suggested to promote optimal clot formation. [36] Nevertheless, there is a lack of data and consensus in many areas, such as the trigger and target fibrinogen levels for various patient groups and clinical scenarios (e.g., in less-than-severe bleeding, in obstetrics or vascular surgery and for various laboratory and viscoelastic POC tests).

Furthermore, uncertainty still exists concerning the optimal timing for both lab sampling and treatment with fibrinogen concentrate and even concerning the adequate dose required to prevent or stop post-CPB bleeding. [9] For instance, a Clauss fibrinogen target of 2.8 g/L has been advocated for pre-emptive substitution. [6,9,16,18] Pre-emptive substitution of fibrinogen concentrate even prior to CPB was also ineffective regarding bleeding and transfusion requirements. [14] Our results and those of others do not provide any evidence to recommend pre-emptive targeting of high fibrinogen levels. [16,25] The early kinetics described in our study also suggest that even the timing of fibrinogen sampling after CPB will influence decision making and dosing if pre-emptive thresholds are used in the absence of significant bleeding. In agreement with our findings, other studies also estimate the recovery rate of fibrinogen at 100 mgL^-1^h^-1^ between the end of CPB and 24 h thereafter in patients without administration of exogenous concentrate, [16] and at 130 mgL^-1^h^-1^ in patients receiving FFP but not fibrinogen concentrate after CPB. [17] Plasma fibrinogen is known to return to normal or even to reach supranormal levels within 24 hours independent of exogenous supplementations. [17,37] Our data show an approximate linearity of spontaneous fibrinogen recovery during these 24 hours.

Nevertheless, the study has some limitations. It is a small, single-center, descriptive series of elective patients. The surgical and perfusion management followed institutional routines. The use of specific perfusion circuits and priming fluids, antifibrinolytics, shed blood recovery, and surgical hemostasis techniques may have influenced the fibrinogen kinetics and thus limited external validity. Additionally, only 38% of patients in our cohort were at high risk of bleeding risk, and the bleeding rate was low. However, data from other groups studying complex cardiovascular surgery with a high bleeding risk have indicated that patients without exogenous fibrinogen replacement experience comparable post-CPB fibrinogen concentrations and bleeding activity than those with substantial substitution of fibrinogen concentrate, and similar recovery of fibrinogen levels that reach or surpass the pre-operative baseline on the first postoperative day. [16,17,25]

In conclusion, this study provides new insights into the fibrinogen substitution practice of cardiac surgery patients. Our exploratory findings suggest that in the absence of active bleeding, low plasma fibrinogen concentration may be tolerated after CPB weaning since plasma fibrinogen levels recover fast and spontaneously at a nearly constant rate. Thus, in our opinion, exogenous fibrinogen substitution may be reserved for patients with active non-surgical bleeding accompanied by laboratory evidence of hypofibrinogenemia. Especially in patients with mild or insignificant post-CPB bleeding, a “wait-and-see” approach appears to be better justified than administration of exogenous fibrinogen based on a presumed bleeding risk and a single-point laboratory measurement. Since our results have been obtained in a small and narrowly defined patient cohort, our study remains descriptive and is not confirmatory. Our findings should therefore be considered only as proof of concept at this time. Our conclusions need further validation and confirmation, e.g., by ongoing and future randomized multicenter trials.
